# Developing Intervention Strategies to Optimise Body Composition in Early Childhood in South Africa

**DOI:** 10.1155/2017/5283457

**Published:** 2017-01-16

**Authors:** Catherine E. Draper, Simone A. Tomaz, Matthew Stone, Trina Hinkley, Rachel A. Jones, Johann Louw, Rhian Twine, Kathleen Kahn, Shane A. Norris

**Affiliations:** ^1^Division of Exercise Science and Sports Medicine, Department of Human Biology, University of Cape Town, Cape Town, South Africa; ^2^MRC/Wits Developmental Pathways for Health Research Unit, Faculty of Health Sciences, University of the Witwatersrand, Johannesburg, South Africa; ^3^Department of Psychology, University of Cape Town, Cape Town, South Africa; ^4^Institute for Physical Activity and Nutrition (IPAN), School of Exercise and Nutrition Sciences, Deakin University, Geelong, VIC, Australia; ^5^Early Start Research Institute, Faculty of Social Sciences, University of Wollongong, Wollongong, NSW, Australia; ^6^MRC/Wits Rural Public Health and Health Transitions Research Unit (Agincourt), School of Public Health, Faculty of Health Sciences, University of the Witwatersrand, Johannesburg, South Africa; ^7^Umeå Centre for Global Health Research, Division of Epidemiology and Global Health, Department of Public Health and Clinical Medicine, Umeå University, Umeå, Sweden; ^8^INDEPTH Network, Accra, Ghana

## Abstract

*Purpose*. The purpose of this research was to collect data to inform intervention strategies to optimise body composition in South African preschool children.* Methods*. Data were collected in urban and rural settings. Weight status, physical activity, and gross motor skill assessments were conducted with 341 3–6-year-old children, and 55 teachers and parents/caregivers participated in focus groups.* Results*. Overweight and obesity were a concern in low-income urban settings (14%), but levels of physical activity and gross motor skills were adequate across all settings. Focus group findings from urban and rural settings indicated that teachers would welcome input on leading activities to promote physical activity and gross motor skill development. Teachers and parents/caregivers were also positive about young children being physically active. Recommendations for potential intervention strategies include a teacher-training component, parent/child activity mornings, and a home-based component for parents/caregivers.* Conclusion*. The findings suggest that an intervention focussed on increasing physical activity and improving gross motor skills per se is largely not required but that contextually relevant physical activity and gross motor skills may still be useful for promoting healthy weight and a vehicle for engaging with teachers and parents/caregivers for promoting other child outcomes, such as cognitive development.

## 1. Introduction

Global levels of overweight and obesity in early childhood have risen dramatically in the last two decades [1], with 76% of overweight children under the age of five years living in low- and middle-income countries (LMICs) [2]. In South Africa 22.9% of 2–5-year-old children are overweight and obese [3]. A wide range of physical and psychosocial health and economic consequences of childhood obesity are well documented [4–6]. This evidence underlines the importance of focusing on a number of young children's key health behaviours, such as physical activity, in order to prevent anticipated trends in obesity [1, 2, 7].

It has been recommended that children of preschool age (approximately 3–5 years old) participate in at least three hours of physical activity of any intensity every day [8] and engage in less than two hours of screen time per day [9]. Higher levels of physical activity in early childhood have been associated with favourable measures of body composition [10–14], positive psychosocial and physical health outcomes [15, 16], and gross motor proficiency in early [17] and later childhood [17–19]. Poor gross motor skills have been linked to overweight and obesity in early childhood [20] and later childhood [18, 21, 22], as well as increased sedentary behaviour (SB) [19]. Sedentary behaviour has also been associated with overweight [10, 11, 23, 24] and poor psychosocial and cognitive outcomes [24, 25] in early childhood.

Very little work has been done on physical activity and gross motor skills with young South African children. Data on physical activity in rural South African children (7–15 years old) showed low levels of moderate- to vigorous-intensity physical activity (MVPA), but a high volume of low-intensity physical activity [26]. It has not been established if similar physical activity levels would be found in younger children and children from urban settings. In South Africa, a study with preschool children from low-income, urban settings showed adequate gross motor skills [27], but it is unclear whether these levels of proficiency would be similar in other South African settings.

In early childhood, children's health behaviours are more open to change [28]. The preschool setting is ideal for the promotion of physical activity, since preschool children spend a significant amount of time at preschool and are responsive to environmental control [7, 28]. For interventions delivered by preschool teachers, the importance of support [29, 30] and effective and practical training for these teachers has been emphasised [29, 31]. Parents should be actively engaged in these interventions [31] in order to improve intervention effectiveness [32], as parents influence children's PA in the early years [33–35]. It has been recommended that early childhood interventions to prevent obesity incorporate behaviour change theory [36–39].

While interventions in the preschool environment are important, there is a need for more high quality family-based intervention studies for this age group [40, 41]. There has also been a call for interventions with a greater intensity of intervention dose [41] and interventions using innovative strategies to enhance effectiveness in early childhood [42]. Furthermore, these strategies need to be tested in LMICs, such as South Africa, since most obesity prevention interventions for early childhood have been designed and implemented in high-income countries (e.g., [31]).

Therefore, the aim of this study was to develop a better understanding of physical activity, sedentary behaviour, and gross motor skills of preschool children in South Africa, as well as factors within the home and preschool environment relating to these behaviours and skills. This was done to prepare for the development of a theory- and evidence-based intervention to optimise body composition in early childhood.

## 2. Methods

### 2.1. Study Approach

The UK Medical Research Council's guidelines on the development and evaluation of complex interventions [43] informed this research, in particular the “development” component. Specific elements of this component included “identifying the evidence base” and “identifying or developing theory” components of the development and evaluation process. Interventions are not only complex in and of themselves, but the context in which they will be applied adds substantially to that complexity. In South Africa, 37% of the population lives in rural areas [44], and 1 in 5 lives in extreme poverty [45]. Furthermore, South Africa has 11 official languages. The development of interventions within LMICs such as South Africa is challenging but must consider different populations residing in different setting (urban and rural) and must be acceptable and feasible where resources (material and human) are scarce.

Ethical approval for this research was obtained from the University of Cape Town Human Research Ethics Committee (HREC REF 237/2012), the University of the Witwatersrand (Wits) Human Research Ethics Committee (Medical) (M140250), and the Mpumalanga Provincial Departments of Health and Education. Written informed consent (parental consent for children) was obtained from all participants.

### 2.2. Study Settings

Data were collected in three settings: a low-income rural, a low-income urban, and a high-income urban setting. Two settings (one low-income and one high-income) were based in Cape Town. The low-income setting in Cape Town was a “township,” with Black African being the predominant ethnicity. Most residents speak Xhosa and the setting has a combination of informal (“shacks”) and formal housing (brick and cement). Common challenges in this community include overcrowding, crime, unemployment, alcohol abuse, and human immunodeficiency virus/acquired immune deficiency syndrome.

The high-income setting was a collection of adjacent suburbs in Cape Town, where residents are predominantly Caucasian and where the population density is substantially reduced (approximately 15 times lower) compared with the low-income Cape Town setting. The area has a number of high quality private and public schools, various private health facilities and services, public parks and green spaces (with children's playgrounds), expensive retailers, and well-serviced amenities. The Medical Research Council/Wits University Rural Public Health and Health Transitions Research Unit (Agincourt) research site in Mpumalanga, a largely rural province in northeast South Africa, was the second low-income site. Being part of the Bushbuckridge subdistrict and comprising 31 geographically distinct villages, the research site is the location of the MRC/Wits-Agincourt Unit's health and sociodemographic surveillance system established in 1992. Agincourt village itself was the specific setting for the formative research and has a population density of ±607 persons per km^2^. The area is characterised by household plots supporting limited subsistence agriculture. Unemployment is widespread, with an estimated 60% of men and increasing numbers of women migrating to more urban areas for work and many households dependent on social grants such as old age pension and child support grants [46, 47]. The predominant ethnicity in Agincourt is also Black African, and the common language is Shangaan.

### 2.3. Study Sample and Recruitment

Preschools were the main point of recruitment for children, teachers, and parents/caregivers (hereafter referred to as parents). In Cape Town, preschools in both the high- and low-income settings were selected using convenience sampling and based on existing contacts. The preschools invited to participate were intentionally diverse to ensure that they were as representative as possible, taking into account geographical location and socioeconomic status at a community level. In Agincourt, recruitment was coordinated through the Stakeholder Relations Office, and its Learning, Information Dissemination and Networking with the Community (LINC) team in the MRC/Wits-Agincourt Unit. There are three preschools in Agincourt village and all were recruited to participate in the study. At the initial stage of the research in Agincourt, it became evident that some preschool-aged children (4–6 years old) had already moved up to Grade R (first year of formal schooling). Therefore, to maximise the sample size, Grade R children from the two primary schools in the village were also recruited and included in the sample of low-income rural children.

Initial telephonic contact was made with the principals of the preschools and primary schools in order to determine their willingness to be involved in the research, followed by a site visit to discuss and explain the research objectives and approach. Information sheets and consent forms pertaining to the child assessments and the focus groups were distributed through parent information sessions and/or at drop-off and pick-up times. Additionally, information sheets and consent forms were sent home with children. The information sessions were generally poorly attended, which necessitated a combination of approaches to facilitate recruitment.

Parents for the focus groups were recruited through parent information sessions. Teachers from the preschools and schools were also approached individually and invited to participate in a focus group. Details of participant numbers and data gathering methods are provided in Table 1.

### 2.4. Data Collection

#### 2.4.1. Assessment of Anthropometrics

Children's height and weight were measured (shoes and heavy clothing removed) using a portable stadiometer (Leicester 214 Transportable Stadiometer; Seca GmbH & Co, Hamburg, Germany) and a calibrated scale (Soehnle 7840 Mediscale Digital; Soehnle Industrial Solutions GmbH, Backnang, Germany). All measurements were taken twice, and an average was taken of the two for analysis. Height and weight measurements were used to calculate Body Mass Index (BMI). The International Obesity Task Force (IOTF) [48] cut-offs were used to classify children as normal weight, overweight, obese, and morbidly obese or thinness. BMI *z*-scores were computed using the WHO AnthroPlus software (http://www.who.int/growthref/tools/en/).

#### 2.4.2. Assessment of Physical Activity and Sedentary Behaviour

Actigraph GT3X+ accelerometers (Actigraph LLC, Pensacola, FL, USA) were used for the objective measurement of physical activity. Accelerometers have been established as a reliable and valid objective measure of physical activity in this age group [49]. The accelerometer was fitted to each child's right hip using an elasticated belt. Children wore the monitors for 24 hours per day over a seven-day period. ActiLife v.6 (ActiLife software; Pensacola, FL, USA) was used to manage the data. Participants' data were included if they had seven hours of valid wear time (excluding sleep time) on three weekdays and one weekend day [50]. Data were recorded in 15-second epochs [49], and non-wear time was defined as 20 minutes of consecutive zeroes and was removed from data [51]. The cut point used for total physical activity was >25 counts per 15 seconds [52].

The Observational System for Recording Physical Activity in Children-Preschool version (OSRAC-P) was used for the direction observation of physical activity and sedentary behaviour. The OSRAC-P is a direct observational system designed to collect data about children's physical activity in preschools and the behavioural and contextual circumstances of these environments [53] and has been used extensively in a number of studies (e.g., [54–56]), including in South Africa [57]. Data for the OSRAC-P were captured electronically on a Google Nexus 7 tablet, using the Open Data Kit (ODK) Collect application (https://opendatakit.org/about/). At each preschool, 10–12 children were selected for observation (as per the OSRAC-P protocol); the first 10 children who arrived and had informed consent from their parent were selected. Thirty observations of each child were recorded, which took around 15 minutes per child. Observations at each school took place between approximately 08:00 and 12:00 and were done by one observer in the rural setting and another observer in all the urban settings. Teachers were asked to continue with the usual daily schedule while the observations took place. As per previous studies [58], for the physical activity intensity categories within the OSRAC-P, “stationary” and “limb movement,” were combined into sedentary behaviour; “slow easy” was referred to as light physical activity, and “moderate” and “fast” were combined into MVPA.

#### 2.4.3. Assessment of Gross Motor Skill Proficiency

The Test for Gross Motor Development-Version 2 (TGMD-2) was used to assess gross motor skill proficiency. This is a valid and reliable criterion-norm referenced test for children aged 3 to 10 years [59]. Standard testing procedures were used, testing took place at the school, and children were tested in groups. Each skill was first demonstrated to the children and then they were given two opportunities to perform the skill. The testing was video-recorded to allow for more accurate scoring [60]. TGMD-2 raw scores, standard scores, and gross motor quotient (GMQ) scores were generated for each child. The GMQ score was used to categorise children into descriptive categories generated by the TGMD-2 normative reference data (based on US norms; no LMIC norms available). These categories are “very superior,” “superior,” “above average,” “average,” “below average,” “poor,” and “very poor” [59].

#### 2.4.4. Preschool Teachers and Parents/Caregivers' Perceptions: Focus Groups

The aim of the focus groups was to obtain perceptions regarding the importance of physical activity for young children and potential barriers associated with physical activity and gross motor skills development for young children. All focus groups in Cape Town took place at the preschools and those in Agincourt took place at the MRC/Wits-Agincourt Unit offices. All discussions were audio-recorded. In Agincourt, focus groups were conducted in the local language (Tsonga), and then translated and transcribed into English by the local fieldworker. This was then followed by a debriefing with the fieldworker, MS, and CD to discuss the transcripts. During the focus groups in Agincourt, MS took notes on the discussion. In Cape Town, focus groups were facilitated by MS, who also took notes during these discussions. Both the fieldworker and student had received training in facilitating focus groups. In one of the communities where Xhosa is the home-language (although many parents are fluent in English as well), a Xhosa fieldworker assisted with conducting the focus group, translating where necessary. These focus groups were transcribed in English by MS.

### 2.5. Data Analysis

#### 2.5.1. Anthropometric, Physical Activity, Sedentary Behaviour, and Gross Motor Skill Data

Mean BMI values and BMI *z*-scores were compared using 3-way ANOVA analyses, between the urban low-income, urban high-income, and rural low-income samples, along with the percentages of children in the different weight status categories. Descriptive statistics were generated to establish time spent in objectively measured total physical activity and to determine compliance with physical activity guidelines for preschool children. While further analyses of objectively measured physical activity data are possible, the analyses presented in this paper are those that can sufficiently inform intervention recommendations.

OSRAC-P data from the ODK Collect application were uploaded automatically to ODK Aggregate and then exported for analysis. Data were calculated as percent of time (%) spent in different physical activity intensities and compared using Chi-squared analyses. For Chi-squared analyses including tables with values less than five in any cell, Fisher's exact test was performed and *p* value reported. For the TGMD-2 data, descriptive statistics were calculated for GMQ scores and also compared using Chi-squared analyses. For the purposes of these analyses, “very poor,” “poor,” and “below average” were combined into one category; “above average,” “superior,” and “very superior” were also combined into one category.

#### 2.5.2. Focus Group Data

Focus group transcripts were analysed thematically using an adaption of Krueger and Casey's [61] classic method of categorising and coding. Transcripts were coded with the assistance of Atlas.ti Qualitative Analysis software. Responses were grouped into multiple categories. Although there is no strict formula for analysing a response, the content was analysed using the following guidelines [61]: frequency, specificity, and emotion. If a specific theme was raised three or more times at different points in the conversation by different participants within or across focus groups, it was given more weight analytically. Responses that provided more specific information regarding potential intervention strategies were noted. Responses that indicated why a certain approach would or would not work were far more valuable if they expressed the perceived reasons why said approach will or will not work. A member of the research team noted strongly emotional, passionate, or enthusiastic responses during the focus groups, and these were taken into account when considering potential intervention strategies.

## 3. Results

### 3.1. Weight Status

Age, BMI, BMI *z*-scores, and weight status of children are presented in Table 2. The majority (69%–76%) of children in all settings had a BMI in the normal range. Overweight and obesity were most prevalent in urban low-income children (14% compared to 4% in the urban high and rural settings). Setting and weight status were significantly related (chi^2^ = 19.7, *p* = 0.002).

### 3.2. Physical Activity and Sedentary Behaviour

#### 3.2.1. Objective Measurement

The mean total PA per day was 462.0 ± 64.4 minutes. This far exceeded the recommendation of 180 minutes of total PA per day for preschool-aged children. All children with valid accelerometry data met the recommendation in terms of weekly average. The compliance of the sample for meeting physical activity recommendations everyday was also very high, with only four children (1.8%) not meeting the physical activity recommendation for all valid days of wear.

#### 3.2.2. Direct Observation

Table 3 presents the percentage of time spent at the different PA intensities as measured by direct observation, as well as time spent inside and outside school buildings. Data from the urban setting have previously been published [57] and are presented here for the purposes of comparison with the rural setting. Children from the rural setting spent more time in MVPA and more time outside when compared to the two urban groups. The differences between settings in proportion of time spent in different intensities were significant (Chi^2^ = 13.27, *p* = 0.01). When the rural children were outside, most of their activity was unstructured, with very little facilitation of physical activity by teachers. In the urban settings, time outside was spread more evenly between structured (including teacher-initiated activities) and unstructured play. In the urban preschools, outdoor space was much more abundant in high-income preschools, and they generally had more outdoor fixed equipment that was generally in better condition, compared to the low-income preschools. In the rural preschools, there was also an abundance of space and sufficient outdoor fixed equipment, but the surface of the play area was generally less well maintained and was mainly dry grass or sand.

### 3.3. Gross Motor Skill Proficiency

Levels of GMS proficiency were generally high, with only 7% of the total sample scoring in the very poor to below average range, as shown in Table 4. The differences between settings in gross motor proficiency were significant (Chi^2^ = 18.53, *p* = 0.001).

### 3.4. Focus Group Findings

Summaries of the focus group findings and selected quotes are presented in Table 5 (perceptions of PA and related issues) and Table 6 (perceived barriers to PA and GMS development).

## 4. Discussion

### 4.1. Summary and Implications of Main Findings

In this study, between 4 and 14% of children were classified as overweight or obese. These levels are lower than the SANHANES-1 [3], which reported from a nationally representative sample that 18.2% were overweight and 4.7% were obese. However, this study identified a similar trend to SANHANES-1 that levels of overweight and obesity were higher amongst urban, especially low-income children in this age group. Furthermore, research in similar low-income settings in South Africa has highlighted the issue of overweight and obesity in young children and has specifically shown that early childhood is a crucial period for predicting obesity in adolescence [62], which emphasises the importance of both the prevention and management of overweight and obesity in early childhood. In the low-income urban setting, rapid social and economic transitions could be contributing to the increase in overweight and obesity amongst children, as well as the burden of noncommunicable diseases in these settings in South Africa [63, 64]. In addition, it has also been shown that noncommunicable diseases disproportionately affect poor people living in urban areas in South Africa [65].

Around a quarter of the high-income and rural children had low BMI for their age (thinness), and this was an unexpected finding. In rural areas, the focus groups highlighted that this might be due to poor nutrition, but it is unclear what would contribute to thinness amongst high-income children. It is important to consider the potentially negative consequences of promoting vigorous physical activity (and hence energy expenditure) in children who have low energy resources as a result of undernutrition. For children in these circumstances, lower intensity physical activity may be more appropriate, and it could still be beneficial for young children [66].

Most of the children included in this study met or exceeded the daily recommendations for physical activity, which is in contrast to research with older children in rural South Africa, where no participants met the recommendation of 60 minutes of moderate-to-vigorous physical activity on most days [26]. It should be noted though that physical activity recommendations for this age group [8] are from four high-income countries, and there is limited evidence, on which these recommendations are based. Therefore, these recommendations may not be the most appropriate level, and they may change as techniques for measuring children's physical activity levels improve. Our findings are also contrary to research from other global settings, which has indicated that the majority of preschool children are not meeting recommendations [9, 67, 68]. Since this study is the first to objectively measure physical activity in South African preschool children, further research needs to be conducted to better understand factors contributing to these high levels of physical activity and what may account for differences with findings from other countries, as well as how these levels may decrease as children start formal schooling.

In this study, more than 90% of children (from all settings) scored within or above the acceptable range for gross motor skills. These confirm previous findings from research on gross motor skills in South African children from low-income settings [27]. However, these data are in contrast to previous research showing that lower socioeconomic status has been associated with lower levels of gross motor proficiency in high-income countries [69, 70]. For example, in low-income settings in the USA, 90% of preschoolers from disadvantaged settings showed developmental delays in their gross motor skills (also using the TGMD-2) [71].

Regarding the observation of physical activity in preschools, the time spent (almost 75%) sedentary in the preschool environment warrants attention. This is especially concerning for low-income urban settings, where over 90% was spent indoors, and there was limited space for outdoor play. Other studies using the OSRAC-P have found similarly high levels of sedentary behaviour (87%–89% of preschool time) [55, 58] and that children tend to be more sedentary when they are inside [55, 72]. Increased time spent outdoors has previously been established as a correlate of preschool children's physical activity [73].

The main findings from the focus groups were that teachers and parents were positive about physical activity and its importance for health and development in early childhood. However, teachers and parents in low-income settings seemed less aware of sedentary behaviour, the potential dangers of high levels of screen time, and the role this plays in children's health and development. In terms of barriers to physical activity, lack of resources, limited capacity (amongst teachers and parents), and safety were the main concerns in low-income settings. Time was the main barrier for teachers and parents from high-income settings.

Given the higher than expected physical activity levels and gross motor skills of the children in all three settings, an intervention specifically focusing on improving these behaviours per se may not be the most appropriate way of optimising body composition in the low-income settings. However, since teachers and parents were positive about physical activity in early childhood, potential intervention strategies could help to address levels of sedentary behaviour in preschools. Intervention strategies could potentially be taken further to use physical activity and gross motor skill activities as a vehicle for the promotion of other child developmental outcomes, such as cognitive development, such as executive function. This is still relevant for optimising body composition in early childhood, since overweight and obesity have been associated with poor executive function in children [42, 74]. This could be done through the provision of educational information and skills training and increasing the motivation of teachers and parents, in line with the theoretical framework proposed earlier. It would be helpful for training to also include nutrition, in order to address the issue of thinness.

### 4.2. Recommendations for Intervention Development

**Figure 1:**
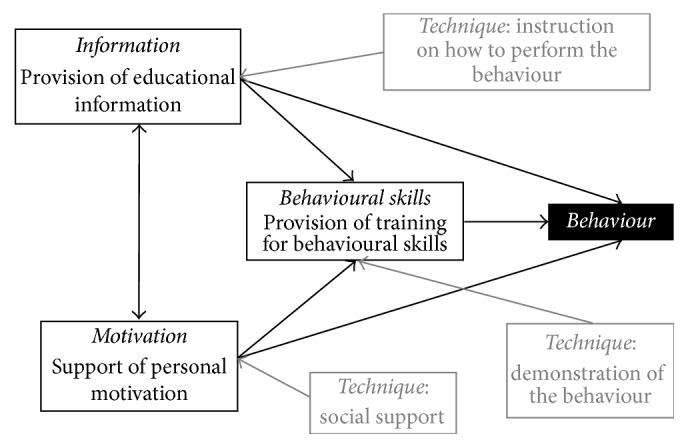
Theoretical framework: Information-Motivational-Behavioural Skills Model.

For the formulation of recommendations for potential intervention strategies, the Behaviour Change Taxonomy [75, 76] was consulted for appropriate theoretically derived behaviour change techniques. From the Behaviour Change Taxonomy [75, 77], the Information-Motivation-Behavioural Skills Model was identified as a feasible and appropriate theoretical grounding for the development of intervention strategies. This model targets three aspects leading to behaviour change: information, behavioural skills, and motivation. The model has been used most frequently in HIV/AIDS intervention strategies but is argued to have potential for broader application for interventions targeting noncommunicable diseases [78], which may include physical activity and nutrition behaviours. The application of the model to this study is presented in Figure 1, and the application of the components of this model to the development of potential intervention strategies is explained in the section that follows.

From the focus group findings, as well as through interactions with the schools and parents, it was evident that further information regarding the benefits of physical activity for young children would be welcomed by teachers and parents (“provision of educational information”) and that teachers would be interested in and would benefit from additional training to lead structured activities. Specifically, information on how activities could be implemented in settings where space and resources were perceived as a barrier could be particularly helpful.

Although the objectively measured physical activity and gross motor skill data indicated that intervention strategies focussed on improving these outcomes were not necessary, the preschool observation data in rural preschools indicated a need for a better balance between structured (specifically teacher-led) activities and free play, since both of these types of activities are beneficial for young children. Focus group findings also indicate that teachers could benefit from provision of training in the behavioural skills required to implement these activities. This could be achieved through the technique of instructing them on how to perform the activities and demonstrating these activities in training sessions or workshops.

Considering the barriers mentioned by parents in the focus groups, it seems that this group would benefit from input on behavioural skills for overcoming these barriers, where possible. In addition, information on the importance of parents in the promotion of healthy behaviours of young children could be a potential area for the provision of information and behavioural skill training, since the focus group findings do not suggest that parents were fully aware of this role, as this particular topic did not feature in the focus group discussions.

For both teachers and parents, the motivation component of the theoretical framework is particularly relevant. The focus group findings indicate that this may be especially pertinent in the low-income settings where resource challenges have the potential to lead to a sense of helplessness regarding their circumstances. Providing social support for these teachers, as well as for parents in their efforts to promote healthy behaviours, would be an important technique to build motivation. For teachers specifically, our interactions confirmed what was suspected: that they are loath to take on any task additional to what is required of them already at school. A programme that has them as primary service delivery agents will have to find a way to motivate them to participate; otherwise such a well-intended effort will simply not be sustainable.

### 4.3. Strengths and Limitations

The main strength of this study is that it has conducted new and informative research on an important target population (preschool children) from a range of settings in South Africa. An additional strength is the use of three different types of settings. These findings will be crucial for the development of an appropriate and feasible intervention to optimise body composition in early childhood. Had an intervention developed for the South African setting purely based on evidence from other countries, it would likely have had a limited effect and may not have been justified in certain settings. Instead, this new research has provided insight into how an intervention, which may still be necessary to prevent overweight and obesity in this age group, could be refocused according to local evidence.

The sample sizes for the studies that make up this formative research are relatively small. However, considering that this work is novel in South Africa, these small-scale studies provide a helpful start for future work in this area. The high levels of activity warrant additional research to investigate the extent to which these findings would be replicated in other settings. However, the relative consistency in patterns across the three settings suggests that these levels may be a reliable reflection. The comparison of gross motor skill results with USA norms could be perceived as a limitation of this study. However, until such time as South African norms are available, the TGMD-2 (with USA norms) remains the best tool for research, particularly since it facilitates comparison with other international studies that have used the same method. Furthermore, the use of a globally recognised measure of gross motor skills could be viewed as a strength of his study.

It was a challenge to obtain good attendance for the focus group sessions. Parents in most of the low-income settings attended well, but parents in the high-income group, as well as at one of the low-income preschools in Cape Town, provided a major challenge. It also proved extremely difficult to arrange a focus group with teachers from high-income preschools. Overall, the process of engaging with parents (through the schools) for this formative work was incredibly challenging. Although attendance was high in the parent focus groups in the low-income settings, we suspect this may have more to do with the transport cost reimbursement and refreshments provided (standard procedure for conducting focus group discussions in the MRC/Wits-Agincourt Unit, therefore this was expected) and less to do with their enthusiasm to participate in the research. In fact, levels of participation in the groups were low, and in Agincourt it was difficult to engage participants in the discussions. It was difficult to get parents to attend meetings (since it was not possible to always offer transport cost reimbursement and refreshments) and elicit responses in the focus groups. Plans for intervention strategies should most certainly take this apparent lack of enthusiasm into consideration, as well as the competing priorities in the various settings. High-income parents and schools may be too busy; urban low-income schools will be most concerned about safe spaces outside their schools; and low-income rural parents want to have something that helps them in the poverty situation they face.

## 5. Conclusion

Somewhat contrary to expectations, we found that the three populations we sampled exhibited adequate levels of overall physical activity and gross motor proficiency and that levels of overweight and obesity were not as high as anticipated. However, our findings still raise concerns about levels of sedentary behaviour in certain settings as well as the thinness of some of the children. Intervention efforts may therefore be better directed at maintaining a healthy weight for the majority of preschool children, which can still be achieved through the promotion of physical activity and gross motor skill development through activities that could also improve cognitive development and the reduction of sedentary behaviour.

Our findings help to serve as a reminder to all service providers of the importance of conducting empirical assessments of the actual conditions that prevail in a specified target population, before an intervention is designed or implemented, a crucial component of the UK MRC framework mentioned earlier. Our findings, although not based on large samples, drew attention to a different set of problems, which may very well benefit from targeted interventions. From our findings, it is apparent that for future intervention efforts it would be important to focus on more than just physical activity, sedentary behaviour, and gross motor skills in order to be salient for preschool teachers and parents and have an impact, particularly in low-income settings.

Furthermore, the data obtained in this study, as well as our experiences in conducting it, show very clearly that implementing any intervention in these three communities would face serious challenges. For a start, intervention content may need to be tailored to the different target groups: the conditions in which these three groups of preschool children find themselves will make it very difficult to have one version of the intended intervention. Furthermore, intervention planners will have to give careful consideration to the actual delivery of the programme activities: how the programme will be organised and how the services will be delivered. Given our experiences with trying to attract parents and teachers to the focus group discussions, we predict that a substantial challenge will be to attract and retain parents and teachers to the activities designed for them. The theoretical framework for the proposed intervention strategies has highlighted the importance of equipping teachers and parents with helpful information and empowering them with appropriate behavioural skills to promote healthy behaviours amongst the children in their care. Very importantly, this framework also emphasises the significance of motivation to act on this information and put into practice these behavioural skills, in a way that leads to a sustained effort and ultimate impact on the children in their care.

## Figures and Tables

**Table 1:** Participant numbers for various data collection methods.

	Urban high-income	Urban low-income	Rural low-income
*Children*			
Objectively measured physical activity and sedentary behaviour	*n* = 30	*n* = 70	*n* = 124
Direct observation of physical activity and sedentary behaviour	*n* = 40 at 4 middle- to high-income preschools; *n* = 1280 observations	*n* = 40 at 4 low-income preschools; *n* = 1201 observations	*n* = 55 at 3 preschools & 2 primary schools (Grade R); *n* = 1693 observations
Gross motor skills	*n* = 46 high-income	*n* = 91 low-income	*n* = 122

*Parents/caregivers and teachers: focus groups*	*n* = 2 (1 high-income teacher group)	*n* = 14, *n* = 3 (2 low-income combined groups)	*n* = 7, *n* = 9 (2 parent groups)
	*n* = 2, *n* = 2 (2 high-income parent groups)	*n* = 6 (1 low-income teacher group)	*n* = 6, *n* = 4 (2 parent groups)

**Table 2:** Weight status of preschool children from urban high-income, urban low-income, and rural low-income settings across South Africa.

	Total (*n* = 258)	Urban high-income (*n* = 46)	Urban low-income (*n* = 91)	Rural (*n* = 122)
Age (years)	5.18 ± 0.70	5.28 ± 0.72	5.35 ± 0.72^a^	5.02 ± 0.64^a^
BMI *z*-scores	−0.05 ± 1.03	−0.25 ± 0.81	0.40 ± 1.05^b^	−0.10 ± 1.02
BMI (kg·m^−2^)	15.46 ± 1.57	15.02 ± 1.11	16.00 ± 1.71^b^	15.22 ± 1.50
Weight status %				
Thinness (low BMI for age)	19	23.92	7.7	25.63
Normal weight	72.09	71.74	75.82	69.42
Overweight	5.81	4.35	10.99	2.48
Obesity & morbid obesity	3.1	0	5.5	2.48

Age and BMI data presented as means ± SD. “a” indicates significant difference between rural and urban low-income children. “b” indicates significant difference between urban low-income children and urban high-income and rural children (all *p* < 0.05).

**Table 3:** OSRAC results of preschool children from urban high-income, urban low-income, and rural low-income settings across South Africa.

	Urban high-income	Urban low-income	Rural
Number of observations	*n* = 1280	*n* = 1201	*n* = 1693
Physical activity intensity %			
MVPA	9	11	22
Light PA	18	16	6
Sedentary	73	73	71^*∗*^
Location %			
Inside	79	93	43
Outside	19	7	55
Transition	2	0	2

^*∗*^“*Can't tell”* coded for 1% in the rural sample.

**Table 4:** TGMD-2 results of preschool children from urban high-income, urban low-income, and rural low-income settings across South Africa.

	Total sample (*n* = 258)	Urban high- income (*n* = 46)	Urban low-income (*n* = 91)	Rural low- income (*n* = 121)
GMQ categories %				
Very poor, poor & average	7	2.2	6.6	9.1
Average	60.5	73.9	71.4	47.1
Above average, superior & very superior	32.7	23.9	22	43.8

**Table 5:** Perceptions of early childhood physical activity and related issues.

Rural	Low-income urban	High-income urban
*Perceptions of early childhood physical activity*		

(i) If a child is not physically active, they are probably ill. (ii) The games they play tell you what career they are likely to have when they are adults. (iii) Children's play copies what they see and experience.	(i) If a child is not physically active, they are probably ill. (ii) Children develop through play. Our children do not have the opportunities to play like the children in richer areas have. (iii) Children's play copies what they see and experience, including on TV and what their parents do.	(i) Physical activity is important to being healthy. Children are regularly monitored for signs of illness. (ii) Physically active play is vital in developing cognitively and socially, as well as developing motor skills. (iii) Children's play copies what they see and experience, including on TV.

*“It helps them they must not get sick simply like coughing. If they running around even the blood can circulate simply in the bodies. When they play you can see that the child is in good health, but if a child sit down for a long time not playing you can also see that the child is sick.”* Parent, rural		
*“They learn through [physically active play], we know that's how they learn and they're not just playing you know. Adults see it as just playing, but it's not playing. It's learning about the world around you, it's learning about how to make a plan when something doesn't work and how to move your body in relation to everything around you. And your friends, social interaction, all those things. And if they're going to formal education too soon, they haven't built up an idea of the world around them, that is strong enough for them to be able to cope with abstract ideas, they haven't built up the vocabulary to cope in classroom so they struggle, because they have huge demands put on them.”* Teacher, high-income urban		
*“The four to five year olds, they generally play quite a lot of fantasy imaginative games and it depends on what their experience is. So if they do watch television they will want to play games like ninja turtles, or if they've watch Pirates of the Caribbean they will play those.”* Teacher, high-income urban		

*Benefits of physical activity*		

(i) Physically active play is important for physical development.	(i) Physically active play is important for physical and mental development.	(i) Physically active play is important for physical, mental, and social development.

*“…it helps with their mental ability and their physical body development, and even in their classes. When they are given a task, or when they come inside, and they play outside then it's good for them, because when they come into the class they will share how they feel about what they did outside.”* Teacher, low-income urban		

*Sedentary behaviour*		

(i) Sedentary behaviour is rare and usually a sign of ill health.	(i) Sedentary behaviour is rare. Children are sometimes kept inside for safety reasons.	(i) Sedentary behaviour is usually a result of technology, but we make sure our children get enough exercise.

*Nutrition*		

(i) The children eat “empty calories” at school. Some children are undernourished. There is a lack of nutritional education.	(i) Diet was not raised as a theme.	(i) Unhealthy food, especially sugar, is considered a big problem for the children.

*“…I find that my kids eat a lot of sweet things, more than I would like. It's so easily accessible, if you go to the shops, it's right at the till. Everything is always (gestures grabbing a snack) and it's so easy to just say ‘get something small, take something small.' So for me it's to get the sugar out of my house.”* Parent, high-income urban		

**Table 6:** Perceived barriers to physical activity and gross motor skills development by location.

Rural	Low-income urban	High-income urban
*Resources*		

(i) Children do not have access to the same facilities as high-SES children. (ii) Poverty is a root cause for many of the issues children face. (iii) Technology was not a barrier to PA, because of lack of access.	(i) Children do not have access to the same facilities as high-SES children. Children often have to travel far for facilities, but this is not the primary barrier. (ii) Poverty is a root cause for many of the issues children face. Money is one of the primary challenges for the school. (iii) Technology was not a barrier to PA, because of lack of access.	(i) We are privileged to have very good facilities. (ii) Some extracurricular activities are expensive, but we can usually afford it. (iii) Technology was potentially the most significant barrier to PA in children.
*“Our children in our community do not know how to swim because they don't have those facilities to learn at. They don't have even the soccer field where they can learn to play. Or netball for the girls. They also don't even know how to play tennis because of the poverty.”* Parent, rural		
*“The sports centre, it's only open when there's a tournament. For the small kids, the preschool children, they don't have the facilities…The only things that are available are for the older kids and older people. So for the small ones, they are not accommodated for.”* Parent, low-income urban		
*“Poverty contributes. Even our municipality does not care about us. If our municipality was interested they would have to build us places where children can be able to play at. And at those places I think they will be playing safe.”* Parent, rural		
*“If anything, I'd say the lure of the screen would be the biggest hindrance to, possibly to physical activity. Possibly the fact that it's more convenient to entertain one's children and easier if you're a child to just sit down and watch something and play with something, than it is to run around.”* Parent, high-income urban		

*Time*		

(i) Time is not the most significant issue for parents.	(i) Time was a problem for some participants. Time is a significant barrier for parents, who usually work.	(i) Time is a significant barrier for parents. Extracurricular activities and domestic help mitigate this.

*“I think the time for parents with kids is very limited in a lot of families.”* – Parent, high-income urban		

*Teacher and/or parent capacity*		

(i) Many parents and teachers do not have the knowledge, training, or energy to properly care for children.	(i) In some settings, parents and teachers do not have the knowledge, training, or energy to properly care for children. (ii) However, in other settings, teachers are self-sufficient and capable, despite their lack of resources.	(i) Teachers are well-trained and capable career teachers. Parents are well educated.

*“You say you might not have space, then we'd take them outside and find space. To run and jump and kick the ball. To me there's always a way out, you need to improvise something if you don't have that...You can't let the child leave the ECD if the child doesn't know how to climb a jungle gym or kick a ball. If you don't have a ball you make one out of paper mache or things like that.”* Teacher, low-income urban		

*Safety*		

(i) Crime is perceived as a real and significant danger to children. (ii) Traffic was dangerous for unsupervised children.	(i) Crime is perceived as a real and significant danger to children. (ii) Traffic was dangerous for unsupervised children.	(i) Crime is perceived as a danger, but not a significant one. (ii) Traffic was dangerous for unsupervised children.
*“It's not safe for our children to go and play outside because there are people who are dangerous these days. They can call your children and promise to give some sweets meanwhile they want to kidnap them or rape them without (being) seen by anybody.”* Teacher, rural		
*“Sometimes if you find that those children are busy playing soccer when they older ones come there they just kick them or chase them.”* Teacher, rural		
*“Yes, I don't even let me two boys, as soon as I fetch them here. They're behind lock and key because I can't let them run on the road at all.”* Parent, low-income urban		
